# Dimensionality and reliability of the Epworth Sleepiness Scale in dental patients referred for oral appliance therapy

**DOI:** 10.22514/jofph.2025.071

**Published:** 2025-12-12

**Authors:** Ksenija Rener-Sitar, Mike T. John, Dennis P. Haley, Anthony J. DiAngelis, Michael J. Howell, Snigdha S. Pusalavidyasagar

**Affiliations:** ^1^Division of Oral Medicine, Diagnosis and Radiology, Department of Diagnostic and Biological Sciences, School of Dentistry, University of Minnesota, Minneapolis, MN 55455, USA; ^2^Department of Prosthetic Dentistry, Division of Dental Medicine, Faculty of Medicine, University of Ljubljana, 1000 Ljubljana, Slovenia; ^3^Center for Prosthetic Dentistry, University Dental Clinics, University Medical Center, 1000 Ljubljana, Slovenia; ^4^Division of Epidemiology & Community Health, School of Public Health, University of Minnesota, Minneapolis, MN 55455, USA; ^5^Department of Dentistry, Hennepin Healthcare, Minneapolis, MN 55414, USA; ^6^Department of Neurology, School of Medicine, University of Minnesota, Minneapolis, MN 55455, USA; ^7^Division of Pulmonary, Allergy, Critical Care and Sleep Medicine, School of Medicine, University of Minnesota, Minneapolis, MN 55455, USA

**Keywords:** Oral appliance, Sleep-disordered breathing, Obstructive sleep apnea, Dimensionality, Reliability, Questionnaire, Excessive daytime sleepiness, Factor analysis, Epworth Sleepiness Scale, Dental sleep medicine

## Abstract

**Background**: There is a limited amount of published research on the dimensionality and reliability of the Epworth Sleepiness Scale (ESS) questionnaire for adult patients referred for oral appliance therapy. This information is crucial for dentists, who often lack objective measures of sleep-disordered breathing (SDB) during titration process of an oral appliance. This study investigated the dimensionality and reliability of ESS scores in adult dental patients with SDB undergoing oral appliance treatment. **Methods**: In 103 dental patients with SDB referred by a physician (mean age: 52.3 ± 13.0 years; 35% female), the dimensionality of the ESS was investigated using exploratory (EFA) and confirmatory factor analyses (CFA) to determine how many scores are needed to characterize the construct. ESS questionnaires were administered twice before treatment. Internal consistency and test-retest reliability were assessed. **Results**: Horn’s parallel analysis suggested a one-factor model. Extracting one factor and standardizing loadings led to strong loadings for all items, ranging from 0.53 to 0.82. The fit indices indicated a good model fit (Comparative Fit Index = 0.999, Root Mean Square Error of Approximation = 0.020, and Standardized Root Mean Square Residual = 0.064). Cronbach’s alpha with 95% confidence interval (CI) was 0.85 (0.82–0.88), indicating strong internal consistency. The intraclass correlation coefficient type 2,1 (95% CI) was 0.86 (0.79–0.90), and the weighted kappa ranged from 0.50 to 0.81. **Conclusions**: In this patient population, the ESS reliably characterizes excessive daytime sleepiness with a single score and appears suitable for individual assessment in dental patients undergoing oral appliance treatment for SDB.

## 1. Introduction

Excessive daytime sleepiness (EDS) remains a major concern in dental sleep medicine, particularly among patients referred for oral appliance therapy. Reliable and valid measurement of EDS, such as with the Epworth Sleepiness Scale (ESS), is important for patient assessment and treatment monitoring in this patient population. The ESS is a frequently used questionnaire to assess self-reported EDS in adult patient populations with sleep-disordered breathing (SDB), including those encountered in dental settings. EDS is a highly prevalent condition, associated with significant morbidity, and results from inadequate sleep, various sleep disorders, medication use, or underlying medical disorders [1]. Although EDS is not always present in patients with SDB [2], it remains one of the diagnostic criteria for obstructive sleep apnea (OSA) [3]. The ESS questionnaire continues to be widely used in clinical dental sleep medicine to quickly assess EDS as a consequence of SDB before and after the initiation of oral appliance treatment [4, 5]. Moreover, the profound consequences of EDS, such as motor vehicle accidents [6] and reduced quality of life [1, 7, 8], further justify the clinical use of ESS.

Usually, eight ESS items with their 0 to 3 response formats are summed to generate a score that characterizes the level of the EDS construct. The underlying summation is that EDS is a unidimensional construct. As emphasized by Hattie [9], the fundamental importance of unidimensionality is that a set of instrument items all measure one construct, which is a basic assumption in measurement theory. The popularity of the ESS suggests that clinicians and researchers feel comfortable with interpreting one ESS summary score, *i.e.*, they assume unidimensionality of this instrument [10].

In at least six studies, unidimensionality of the ESS, derived from exploratory factor analysis (EFA), was confirmed [11, 12, 13, 14, 15, 16]. In contrast, Smith *et al*. [16] used confirmatory factor analysis (CFA) in Australian patients with OSA and found that the one-factor model did not adequately fit the data. Similarly, other studies have reported two-factor structures in different populations, including obstetric patients [17], multinational college student samples [18], English and German language versions [19], large Korean shift worker populations [20], Taiwanese older adults [21], and the Bangla language ESS version [22]. Additionally, a three-factor structure was found in a sample of patients with OSA and a community sample from Western Australia [23]. A systematic review in 2014 concluded that dimensionality of the ESS remains unclear [24], and more recent studies continue to show variable results across different populations [25, 26]. Overall, studies suggest that the dimensionality and scoring structure of the ESS may vary depending on the study population [27].

There is limited published literature on dimensionality and reliability of the ESS questionnaire for adult dental patients referred for oral appliance therapy. However, this information is essential for dentists who often lack objective measures of SDB severity during oral appliance titration [7]. These patients represent a distinct subgroup, typically showing different clinical profiles compared to general sleep clinic populations, including milder OSA severity and specific anatomical features, such as distal occlusion (Angle Class II), that may respond better to oral appliance therapy than to continuous positive airway pressure (CPAP). Therefore, we aimed to investigate the dimensionality and reliability of the ESS questionnaire in a convenience sample of dental patients undergoing oral appliance treatment for SDB.

## 2. Materials and methods

### 2.1 Patient population

Dental records from adult patients referred for oral appliance treatment for SDB to the Temporomandibular Disorder, Orofacial Pain, Oral Medicine, and Dental Sleep Medicine Clinic at the University of Minnesota, Minneapolis, MN, USA (N = 61), and the Dental & Oral Surgery Clinic, Hennepin County Medical Center, Minneapolis, MN, USA (N = 42), who completed the ESS questionnaires twice before treatment, were reviewed between 23 November 2011 and 31 January 2013. Patients completed the first ESS at the baseline evaluation with the dentist (*i.e.*, test) and the second ESS at their next appointment, before the initiation of oral appliance treatment (*i.e.*, retest). The same dentist performed both ESS administrations, and no oral treatment had been initiated during the test-retest period. Demographic and polysomnographic data were also extracted from dental records and de-identified before statistical analysis. Inclusion criteria were adult patients (≥18 years) referred by a sleep physician for oral appliance therapy to manage SDB, with two baseline ESS questionnaires completed. Exclusion criteria were patients ineligible for oral appliance fabrication due to ongoing or planned dental treatment in the near future.

### 2.2 Outcome measures

The ESS is a self-reported, brief instrument that assesses the degree of EDS during routine daily tasks. Prior research suggests that ESS scores reflect a stable characteristic of EDS over time, unless an intervention occurs [25, 26, 28]. It was introduced in 1991 by Johns [29], Epworth Hospital, Melbourne, Australia, for patients with OSA, periodic limb movement disorders, narcolepsy, idiopathic hypersomnia, and other miscellaneous disorders. The instrument contains eight items, usually taking two to three minutes to complete. Individuals are asked how likely they are to doze off or fall asleep in different and frequently encountered sleep-inducing daily situations. They respond on a 0 to 3 numeric rating scale (0 points—“would never doze”, up to 3 points—“high chance of dozing”). These eight items are combined into a summary score ranging from 0 to 24 points (*i.e.*, the highest score when a patient has a high chance of dozing in every situation out of eight). The most commonly used cutoff score to detect EDS is 10 points or higher [30]. A score greater than 16 points indicates a high level of EDS [29]. Patients with moderate to severe OSA, narcolepsy, and idiopathic hypersomnolence usually score high [31, 32]. However, a higher ESS does not necessarily indicate a specific sleep disorder diagnosis [30].

### 2.3 Statistical analysis

#### 2.3.1 ESS item analysis

Mean ESS with 95% confidence interval (CI) for the mean, along with median, minimum, and maximum scores, were computed for each ESS item and the ESS summary score, separately for the test and the retest ESS administration before the insertion of the oral appliance. The Kolmogorov-Smirnov (KS) test was used to assess the normality of the test-retest time interval. Because the test-retest interval was not strictly standardized due to variability in patient availability, we also analyzed the correlation between the test-retest interval and changes in ESS total scores. This additional analysis helped confirm that variability in the timing of assessments did not significantly affect the results.

The ESS, demographic, and polysomnographic data were retrieved from dental records. Missing values existed for demographic and polysomnographic data, which were handled using multiple imputations with fully conditional specification (FCS) in SPSS® v.29 (IBM Corporation, Armonk, NY, USA). Twenty imputed datasets were generated based on a set of available demographic and polysomnographic variables. The FCS method was chosen due to the arbitrary (non-monotone) pattern of missingness and presence of both continuous and categorical variables. Pooled estimates across the 20 imputations were used for descriptive statistics.

#### 2.3.2 Dimensionality

Dimensionality assessment was conducted using a stepwise approach. First, in the test ESS data set, polychoric correlations among the ESS items were inspected to identify patterns of moderate or strong correlations (*i.e.*, 0.50–0.89) that could correspond to underlying factors [33]. Communalities for each ESS item were calculated as squared factor loadings, indicating the proportion of variance explained by the latent factor(s). These communalities are also equal to one minus a unique (residual) variance of each item. Subsequently, EFA was conducted on the polychoric correlation matrix using the iterated principal factor method. Eigenvalues were plotted using Horn’s parallel analysis to determine the number of factors to retain.

The factor structure identified in the EFA was afterwards evaluated using the CFA in the retest data set, *i.e.*, data from the second baseline assessment. CFA was performed using diagonally weighted least squares estimation with ordinal indicators, where polychoric correlations were estimated internally. The latent factor was scaled to have a mean of 0 and a variance of 1. Standardized factor loadings were also reported. To evaluate model fit, we used a set of indices suggested by Kline [34], including the Root Mean Square Error of Approximation (RMSEA), Comparative Fit Index (CFI), and Standardized Root Mean Square Residual (SRMR). Commonly applied guidelines for good model fit propose that CFI ≥0.95, RMSEA ≤0.06, and SRMR ≤0.08 [35], while RMSEA values ≥0.1 indicate poor fit [36]. Recently, dynamic fit index cutoffs were introduced in 2023 [37]. Based on the results from Goretzko *et al*. [38], the average dynamic thresholds for one-factor CFA models that confirm good model fit are CFI ≥0.973, RMSEA ≤0.053, and SRMR ≤0.050. No dynamic thresholds have been proposed for Tucker–Lewis Index (TLI) or Goodness of Fit Index (GFI), and their use has become limited in recent applications. For this reason, we excluded them from our analysis. Finally, all factor analytic results were synthesized to determine how many factors characterize the construct of EDS. All dimensionality computations were performed in R v.4.5.1 [39] using the psych [40] and lavaan packages [41].

#### 2.3.3 Reliability

Internal consistency was evaluated with Cronbach’s alpha [42]. Acceptable values of alpha range from 0.70 to 0.95, but the maximum recommended value is 0.90 [43]. Values above 0.90 suggest some items are redundant [43]. Average inter-item correlations were also computed. An average inter-item correlation between 0.15 and 0.20 is considered satisfactory for scales that measure a broader characteristic, while values between 0.40 and 0.50 are necessary for scales with narrower characteristics [44].

Test-retest reliability of the ESS summary scores was investigated using the intraclass correlation coefficient (ICC), which was calculated using a two-way random-effects model, treating the cases as a random factor, according to Shrout and Fleiss’s ICC model type 2,1 [45]. The ICC indicates an excellent agreement if it is above 0.75, a good agreement for values between 0.6 and 0.74, a fair to moderate agreement for values between 0.4 and 0.59, and a poor agreement if ICC is below 0.4 [46]. Another measure of test-retest reliability for the eight ESS items was computed using the weighted kappa statistic, which is usually utilized for short ordinal scales. For kappa, values of less than 0.4 indicate a slight agreement; between 0.41 and 0.6, a moderate agreement; 0.61 and 0.8, a substantial agreement; and above 0.8, almost perfect agreement [47]. Statistical significance of kappa values was assessed using *Z*-tests, which determine whether agreements differ significantly from chance.

The “Limits of Agreement” (LoA) around a mean difference were calculated as 1.96 times the standard deviation of the differences, according to the method by Bland and Altman [48]. If the 95% CI for the mean of differences excludes zero, this indicates a statistically significant difference between the test and retest data. Agreement between the first and second EDS assessments was also visually inspected with a Bland and Altman plot [48].

## 3. Results

### 3.1 Participants’ characteristics and severity of ESS item impairment

The following demographic and polysomnographic data were extracted from 103 dental records and were available for the majority of participants, except for neck circumference: age (n = 100; 97%), gender (n = 101; 98%), body mass index (BMI; n = 90; 87%), neck circumference (n = 47; 46%), apnea-hypopnea index (AHI; n = 67; 65%), and respiratory disturbance index (RDI; n = 58; 56%). Based on multiple imputation (20 datasets), pooled estimates for the full sample of 103 participants revealed a mean age of 52.3 ± 13.0 years (range: 21–84), with 67 participants (65%) identified as male. The mean BMI was 30.6 ± 4.8 kg/m^2^, mean neck circumference was 40.5 ± 3.5 cm, mean AHI was 15.9 ± 13.0 events per hour (range: 0.3–80.7), and mean RDI was 28.2 ± 16.5 events per hour (range: 7.2–126.7).

No ESS item scores in the first or second administration were missing. The median time between the two baseline ESS administrations was 34 days (range: 12–247 days), present for all 103 participants and not normally distributed (KS statistic 0.227, *p *< 0.001). The association between test-retest intervals and ESS score differences between the first and second administration was assessed by the Spearman correlation coefficient, which was 0.045 (*p* = 0.650), indicating no statistically significant association.

Descriptive statistics of ESS summary scores for test and retest administrations presented for participants with SDB (N = 103) and gender and age subgroups are shown in Table 1. The median value of the ESS summary score in the second administration was slightly smaller than in the first administration, 9 points and 10 points, respectively. The mean difference between the two ESS administrations was 0.18 (95% CI: −0.33–0.70). Table 1 also displays the number and proportion of participants with an ESS summary score of 10 or higher.

**Table 1.** Descriptive statistics for Epworth Sleepiness Scale summary scores at test and retest.

Group/subgroup		N (%)	Test ESS summary score					Retest ESS summary score				
			Mean	SD	95% CI	Min–max	N (%) ESS ≥10	Mean	SD	95% CI	Min–max	N (%) ESS ≥10
All SDB cases		103 (100)	10	4.9	9.0–10.9	1–23	52 (50.5)	9.8	4.7	8.8–10.7	2–20	49 (47.6)
Gender Subgroups												
	Female SDB cases	36 (35.0)	11.2	4.6	9.6–12.7	3–22	24 (66.7)	10.7	4.8	9.1–12.3	2–19	20 (55.6)
	Male SDB cases	67 (65.0)	9.3	5.0	8.1–10.5	1–23	28 (41.8)	9.3	4.7	8.1–10.4	2–20	29 (43.3)
Age Subgroups												
	Young- and Middle-Aged Adults (20–54 yr)	55 (53.4)	10.1	5.2	8.7–11.5	1–23	29 (52.7)	9.9	4.6	8.7–11.2	2–20	27 (49.1)
	Older Adults and Elderly (55+ yr)	48 (46.6)	9.8	4.7	8.4–11.2	3–22	23 (47.9)	9.6	4.9	8.2–11.0	2–20	22 (45.8)

*ESS: Epworth Sleepiness Scale; Test: first administration of the ESS questionnaire; Retest: second administration of the ESS questionnaire; N: number of participants; SD: standard deviation; CI: confidence interval; Min–max: minimum to maximum value; SDB: sleep-disordered breathing.*

Means of individual ESS items ranged from 0.3 to 2.3 points in the test administration and from 0.2 to 2.3 points in the retest administration. Descriptive statistics for individual items from the first and second assessments are shown in Table 2.

**Table 2.** Descriptive statistics for Epworth Sleepiness Scale items at both assessments.

ESS item	Test ESS item score			Retest ESS item score		
	Mean (95% CI)	Median	Min–max	Mean (95% CI)	Median	Min–max
1	1.8 (1.6–2.0)	2	0–3	1.7 (1.5–1.9)	2	0–3
2	1.6 (1.4–1.8)	2	0–3	1.6 (1.4–1.8)	2	0–3
3	1.0 (0.8–1.1)	1	0–3	0.8 (0.7–1.0)	1	0–3
4	1.6 (1.4–1.9)	2	0–3	1.6 (1.4–1.8)	2	0–3
5	2.3 (2.1–2.5)	2	0–3	2.3 (2.1–2.5)	3	0–3
6	0.3 (0.2–0.4)	0	0–3	0.4 (0.2–0.5)	0	0–3
7	1.2 (1.0–1.3)	1	0–3	1.2 (1.0–1.3)	1	0–3
8	0.3 (0.2–0.4)	0	0–3	0.2 (0.1–0.3)	0	0–2

*ESS: Epworth Sleepiness Scale; CI: confidence interval; Min–max: minimum to maximum value.*

### 3.2 Factor analysis

The polychoric correlation matrices for the eight ESS items in patients with SDB (N = 103) are shown separately for the test ESS data, also used for the EFA (lower left triangle), and the retest ESS data, which was also used for the CFA (upper right triangle) in Table 3. Communalities were manually added on the table’s diagonal. Polychoric correlation coefficients among the eight ESS items varied between 0.21 and 0.71 in the test ESS data set, which was used for EFA, and among the first seven items between 0.29 and 0.73 in the retest ESS data set, used for CFA purposes. Polychoric correlation coefficients were not computed for item 8 in the retest data because there were no observations in the response category 3. Communalities ranged from 0.28 to 0.75 for EFA and from 0.28 to 0.67 for CFA (values on the diagonal in Table 3).

**Table 3.** Polychoric correlation matrix and communalities (on the diagonal) for the eight Epworth Sleepiness Scale items.

ESS item	1	2	3	4	5	6	7	8*
1	0.48/0.60	0.71	0.40	0.52	0.42	0.64	0.62	n.a.
2	0.56	0.53/0.67	0.54	0.61	0.32	0.65	0.65	n.a.
3	0.53	0.46	0.57/0.58	0.73	0.46	0.57	0.64	n.a.
4	0.55	0.49	0.69	0.67/0.63	0.52	0.55	0.57	n.a.
5	0.29	0.21	0.34	0.50	0.28/0.28	0.29	0.41	n.a.
6	0.60	0.66	0.59	0.54	0.49	0.63/0.63	0.66	n.a.
7	0.55	0.69	0.65	0.71	0.53	0.61	0.75/0.64	n.a.
8	0.53	0.57	0.54	0.54	0.35	0.70	0.53	0.55/n.a.

ESS: Epworth Sleepiness Scale; n.a.: not applicable; *Polychoric correlations were not computed due to limited variation in response categories.

Horn’s parallel analysis (Fig. 1) supported a one-factor solution, suggesting that EDS, as measured by the ESS, is a unidimensional construct in this patient population. Extracting one factor and standardizing the loadings yielded strong loadings across all items (Fig. 2). The standardized loadings for all eight ESS items ranged from 0.53 to 0.82. The CFA model of the ESS demonstrated a good fit as indicated by multiple indices: CFI = 0.999, RMSEA = 0.020, and SRMR = 0.064.

**Fig. 1.** Horn’s parallel analysis graph. The plot displays eigenvalues (Ev) from the unadjusted (red) and adjusted actual data (black). Only the first adjusted Ev exceeds the corresponding random Ev, which supports the retention of a single factor. Average Evs from randomly generated data are shown in blue.

**Fig. 2.** Confirmatory factor analysis of the Epworth Sleepiness Scale (ESS) unidimensional model. The path diagram displays standardized factor loadings, which are shown next to the arrows. The oval form in the diagram represents the latent variable, and the rectangles represent the eight items of the ESS instrument. Numbers next to error terms (*i.e.*, the Greek letter epsilon or ε) represent error variances, that is, the portion of item variance not explained by the latent factor. ESS: Epworth Sleepiness Scale.

### 3.3 Reliability

Internal consistency statistics were calculated separately for the test and retest data (Table 4). The average inter-item correlation was 0.32. Test-retest reliability for the eight ESS items is also presented in Table 4, in which reliability analyses are reported for the total sample and stratified by gender and age subgroups. Ranges of item-total correlations are also displayed in Table 4. Test-retest reliability was evaluated with ICC type 2,1 with 95% CIs, and these results are also shown in Table 4. Test-retest agreements over time were further assessed by calculating the lower and upper Limits of Agreement (LoA) according to Bland and Altman, which are shown in the last column of Table 4.

**Table 4.** Reliability and test-retest agreement of Epworth Sleepiness Scale scores in all participants and gender and age subgroups.

Group/subgroup		Test ESS		Retest ESS		ICC(2,1) (95% CI)	Lower–upper LoA
		Cronbach’s α (95% CI)	Range of item-total correlations	Cronbach’s α (95% CI)	Range of item-total correlations
All SDB cases		0.85 (0.82–0.88)	0.40–0.75	0.84 (0.81–0.87)	0.39–0.68	0.86 (0.79–0.90)	−4.9–5.3
Gender Subgroups							
	Female SDB cases	0.79 (0.72–0.86)	0.05–0.69	0.83 (0.77–0.89)	0.16–0.87	0.79 (0.63–0.89)	−5.4–6.4
	Male SDB cases	0.87 (0.84–0.90)	0.50–0.78	0.85 (0.81–0.89)	0.42–0.71	0.88 (0.81–0.93)	−4.6–4.7
Age Subgroups							
	Young- and Middle-Aged Adults (20–54 yr)	0.86 (0.82–0.90)	0.44–0.82	0.84 (0.80–0.88)	0.30–0.72	0.87 (0.79–0.92)	−4.8–5.1
	Older Adults and Elderly (55+)	0.82 (0.77–0.87)	0.32–0.68	0.85 (0.81–0.89)	0.41–0.76	0.84 (0.73–0.91)	−5.2–5.6

*ESS: Epworth Sleepiness Scale; Test: first administration of the ESS questionnaire; Retest: second administration of the ESS questionnaire; CI: confidence interval; ICC(2,1): Shrout and Fleiss’s intraclass correlation coefficient, model 2,1; Lower–upper LoA: lower and upper limits of agreement, according to Bland and Altman; SDB: sleep-disordered breathing.*

Agreements between both assessments for the individual ESS items ranged from 93% to 97%, and weighted kappa statistics ranged from 0.50 to 0.81, which were statistically significantly greater than zero, as indicated by *Z* statistics ranging from 5.21 to 8.23 (all *p *< 0.001; Table 5).

**Table 5.** Intra-rater reliability of Epworth Sleepiness Scale items between the test and retest assessment.

ESS item	Agreement (%)	Expected agreement (%)	Weighted kappa	SE	*Z*	Prob>*Z*
1	93.4	79.4	0.680	0.098	6.96	<0.001
2	94.4	82.5	0.680	0.098	6.90	<0.001
3	94.2	81.2	0.690	0.097	7.11	<0.001
4	95.0	74.0	0.809	0.098	8.23	<0.001
5	94.4	83.8	0.654	0.098	6.69	<0.001
6	96.8	90.6	0.657	0.097	6.74	<0.001
7	93.3	79.5	0.673	0.099	6.83	<0.001
8	97.0	94.0	0.500	0.096	5.21	<0.001

*ESS: Epworth Sleepiness Scale; Agreement: percentage of exact agreement between the first and second administrations; Expected agreement: agreement expected by chance; Weighted kappa: measure of agreement corrected for chance with weights for partial agreement; SE: standard error; Z: Z-test statistic to test the null hypothesis about zero agreement; Prob>Z: probability associated with the Z statistic.*

Differences in ESS summary scores between the first and second administrations against the mean ESS scores for each participant are displayed as a Bland-Altman plot in Fig. 3. Most of the differences clustered around zero, with most values falling within a range of approximately ±2 points, although a few outliers were observed.

**Fig. 3.** Bland-Altman plot. It shows the differences in ESS summary scores between two assessments (vertical axis) against the mean ESS summary scores (horizontal axis) for each participant. ESS: Epworth Sleepiness Scale.

## 4. Discussion

This study evaluated the dimensionality and reliability of the ESS in dental sleep medicine patients who were referred for oral appliance therapy. Our results supported a unidimensional factor structure of the ESS, with EFA indicating one underlying factor, and CFA demonstrating a good model fit, according to conventional and also recently proposed dynamic fit index cutoffs. Our results align with previous studies that support a unidimensional structure of the ESS [11, 12, 13, 14, 15, 16] rather than those proposing two- or three-dimensional ESS structures [16, 17, 18, 19, 20, 21, 22, 23], suggesting that the dimensionality of ESS may vary depending on the population studied. In our sample of dental sleep medicine patients referred for oral appliance therapy, the unidimensionality assumption was confirmed, supporting the continued use of a single ESS summary score to represent EDS in this patient population.

The results of this study indicate that both dentists and physicians can trust ESS scores because the psychometric properties of this scale proved adequate in this patient population. This finding is clinically relevant because this is an important and unique patient population. These patients are referred not only for treating OSA but also for upper airway resistance syndrome, a subtype of OSA, and for primary snoring [49]. This population also differs because the referral process to a dental office differs from that of a medical clinic. CPAP is a standard medical therapy for OSA that provides objective efficacy data on treatment, but dentists rely more on subjective measures of treatment success. The EDS construct is particularly important for dentists because, for most clinical cases with SDB, this construct represents an essential treatment outcome.

The average ESS score for both administrations was around 10, which is also the cutoff score indicating mild sleepiness. This is an interesting finding because these patients, who were referred to a dentist for treatment with an oral appliance, predominantly had only mild to moderate OSA, and previous studies have already confirmed that there is little correlation between OSA severity and EDS [50]. We performed a *post hoc* subgroup analysis to explore whether psychometric properties of the ESS differed by gender or age group. While the ESS demonstrated acceptable reliability across all subgroups, some variation was observed. For example, test-retest reliability for the total ESS score was slightly higher in males than in females. It was also somewhat higher among younger and middle-aged adults versus older adults and elderly participants. Internal consistency results followed a similar pattern, *i.e.*, the Cronbach’s alpha ranged from 0.79 in females to 0.87 in males. Although these differences are not large, they may reflect some variations in how EDS is self-perceived within these subgroups. Our female participants showed slightly higher mean ESS scores than the males. Similarly, other studies have also confirmed gender-specific differences in self-reported EDS, as assessed with the ESS questionnaire, where females reported significantly higher total ESS scores than males [51, 52].

Although we lacked sufficient data on nocturnal oxygen desaturation and arousal index, which are recognized contributors to sleepiness, the observed variability in ESS scores probably reflects differences in physiological parameters, such as hypoxic burden. Our sample’s wide range of ESS scores (*i.e.*, 1–23) highlights the variability in subjective daytime sleepiness among patients referred for oral appliance therapy for SDB. While an ESS score of 10 or higher is often used as a threshold for clinically relevant EDS, our results show that not all patients with SDB report EDS, which aligns with previous studies [52], and this can also impact treatment decisions. For instance, patients with lower ESS scores (*i.e.*, without EDS) might be less motivated to regularly use an oral appliance despite objective evidence of SDB, whereas those with higher ESS scores may experience a greater perceived benefit from adhering to this therapy. Therefore, ESS variability may predict patient adherence to this therapy in dental sleep medicine settings [53]. In other similar studies assessing EDS with the ESS in patients with mild to moderate OSA, the average ESS scores ranged from 9.5 [29] to 12 [54] for mild OSA and from 11.5 [29] to 13 [29, 54] for moderate OSA. In another study, a mean ESS score of 10.7 was reported for mild to moderate OSA [55], which is similar to our study results. These results indicate that our study’s findings regarding OSA disease severity and self-reported EDS are consistent with other patient populations where oral appliances are a treatment option, suggesting that the dimensionality and related findings are valid for these populations.

To evaluate the structure of the ESS, we conducted a factor analysis to explore its underlying dimensionality. This methodology is appropriate for assessing whether the observed item responses reflect a single latent construct, *i.e.*, EDS, within this clinical population. This is also relevant in patients with SDB referred for oral appliance therapy, because identifying the scale’s factor structure ensures valid interpretation and use of ESS scores in dental sleep medicine contexts. Our assessment of dimensionality proceeded in a stepwise fashion, from an exploratory to a confirmatory phase. The EFA and visual inspection of the plots of actual versus random eigenvalues both revealed a single common latent factor that explained item responses. All results of the EFA favored unidimensionality, which was confirmed by all the fit indices of the CFA based on guideline recommendations, with values well within traditionally accepted cutoffs [35] and mostly exceeding the dynamic cutoffs recently proposed by Goretzko *et al*. [38]. The SRMR of 0.064 was much below the traditional threshold (≤0.08) and, although slightly above the dynamic recommendation (≤0.050), still indicated an acceptable fit. Overall, our CFA results supported a good fit of the model to our data. In general, a questionnaire’s dimensionality is ideally constant across populations. While the ESS does not appear to be unidimensional in all populations, our study provides additional evidence that a one-factor model is adequate in patients referred by physicians for oral appliance treatment for SDB.

Items 6 and 8 raised concerns in previous studies of ESS dimensionality because low factor loadings have been reported for these two items [11, 12], and in one study, better-fit indices were observed when these two items were excluded [16]. A concern has been raised that these two items may be less than optimal for assessing the construct of EDS, as there is a smaller chance of dozing while talking to someone or being stopped in traffic, as described in items 6 and 8, respectively [24]. Nevertheless, we were unable to confirm those previous findings. Our CFA produced high loadings for all eight ESS items, including items 6 and 8 (Fig. 2). Still, ESS items inquire about common daily routines associated with EDS, and their relevance may vary across different cultures. For example, the first ESS item refers to reading, which is a daily activity that may not be very common in cultures where the literacy level is low. Similarly, the second ESS item, which refers to watching TV, is not a typical daily routine for many people in developed countries, because they prefer to follow the news and watch movies through laptops and smartphones. The seventh ESS item, which mentions alcohol consumption, may also not be appropriate in cultures where alcohol use is generally avoided, such as in Muslim cultures. Also, the fourth and eighth ESS items, which address falling asleep while traveling in a car, may not be applicable in regions where cars are not a common mode of transportation.

The internal consistency of the ESS, as assessed with Cronbach’s alpha, was very good (0.85 for the test and 0.84 for the retest data). In a systematic review about psychometric properties of the ESS, Cronbach’s alpha ranged from 0.7 to 0.9 [24]. In a 2023 meta-analysis by Gonçalves *et al*. [56], it was also concluded that the internal consistency of the ESS, and specifically Cronbach’s alpha, was good (approximately 0.82), but considerable heterogeneity was observed among studies. They reported that factors significantly affecting ESS reliability included the study setting, with clinical populations showing higher internal consistency (alpha = 0.84) compared to community samples (alpha = 0.78). They also noted that studies conducted in Asia had slightly higher Cronbach’s alpha values for the ESS scale (0.838) compared to Europe (0.798), North America (0.82), and South America (0.82). However, they confirmed that other examined variables, such as publication date, language, participants’ sex, age, and risk of bias, did not significantly explain variability in reliability scores. Therefore, interpreting the lower CI limit for Cronbach’s alpha of 0.81, our results suggest that the reliability of ESS scores in SDB patients referred to the dentist may be higher than in other SDB populations where the ESS is also used for evaluating EDS.

The test-retest reliability of the ESS total score was excellent, with an ICC of 0.86 (95% CI: 0.79–0.90), which indicated a good ESS scale stability over time. For individual ESS items, weighted kappa values ranged from 0.500 to 0.809, which means moderate to substantial agreement between test-retest assessments. The lowest agreement between test and retest was observed for the eighth ESS item (kappa = 0.500), while the highest one was for the fourth ESS item (kappa = 0.809). Even though some ESS items showed more variability over time, the overall scale demonstrated excellent temporal reliability. We assessed the agreement between the two ESS administrations with the Bland-Altman method, which is a widely used approach. However, the 95% LoA, according to Bland and Altman, was −4.9 to 5.3 (Table 4), indicating that individual differences in EDS exceed the minimal clinically important difference for the ESS, which is estimated to be between 2 and 3 points [57]. The Bland-Altman plot (Fig. 3) also confirmed that the differences between the first and second ESS mostly fell within the −2 and +2 points, although some outliers were present. These outliers may be a consequence of subjective variations in EDS reporting, differences in sleep behavior and sleep quality, changes in health status or lifestyle, or potential recall bias when completing the questionnaire. However, Spearman correlation analysis revealed no statistically significant association between the length of the test-retest interval and the ESS test-retest score differences.

The strength of this study is that sleep physicians evaluated all patients to confirm the presence of SDB before they referred them to dentists for oral appliance therapy. Additionally, previous studies have confirmed that the relationship between BMI and ESS scores is independent of AHI and concluded that a higher BMI is associated with increased EDS due to mechanisms beyond OSA [52]. In this context, our Minnesotan study population provided a valuable cohort for analysis because the average BMI in this U.S. state is below the average for the Midwest region, and the overall obesity rate is lower than in the most affected U.S. states [58]. Therefore, our Minnesota population introduced less obesity-related bias when analyzing SDB outcomes.

This study also has limitations. We did not collect data on patients’ medical comorbidities or their medication use since our primary aim was to explore the dimensionality and reliability of the ESS in patients referred for oral appliance therapy. However, including such information would have provided a more comprehensive characterization of our study sample. Also, the period between test and retest baseline administrations of the ESS varied among participants and was, on average, six weeks. A more standardized test-retest period would have been desirable, likely providing even more reliable results because longer intervals may have allowed changes in the construct. Despite the relatively long interval between the two baseline assessments, the construct of EDS remained stable, providing evidence that EDS was consistent even over several weeks in this patient sample. In contrast, Nguyen *et al*. [59] reported high variability in ESS scores when sequentially administering the ESS in a population with possible OSA, with an average interval of 71 ± 92 days, roughly twice as long as in our study. While temporal stability over periods longer than two months may be questionable, our findings indicate that EDS remains stable for a median time of over one month in patients undergoing oral appliance treatment. We did not set strict retest interval criteria due to variability in patient availability and clinical follow-up schedules, but all intervals were recorded and statistically analyzed. The relationship between test-retest interval and changes in ESS scores was not statistically significant, thereby suggesting minimal impact. All 103 participants’ data did not meaningfully affect the results, supported by a high ICC of 0.86 (95% CI: 0.79–0.90) and a relatively narrow CI. Agreement for individual ESS items ranged from 93% to 97%, and weighted kappa values ranged from 0.50 to 0.81, indicating moderate to substantial item-level agreement. The small mean difference between ESS administrations (0.18; 95% CI: −0.33 to 0.70) further confirmed that test-retest reliability was stable despite interval variability. Since this was a clinical, not experimental study, we included all participants to reflect real-world conditions. Excluding outliers would not have changed the conclusions and might have introduced selection bias. Although a sensitivity analysis excluding outliers revealed no meaningful change, a more uniform retest interval closer to our median time of 34 days might have reduced variability and improved the precision of reliability estimates.

Lastly, for sample size estimation in our study, we considered the number of ESS items, the number of underlying factors, and the strength of the factor loadings. Recent research suggests that when factor loadings exceed 0.50, samples as small as 100 can yield reliable results, particularly for unidimensional models [60, 61]. Given that the ESS consists of only eight items and our CFA yielded factor loadings ranging from 0.53 to 0.82, our sample size was within the acceptable range for robust factor analysis. Furthermore, our CFA results demonstrated a good model fit, which confirms the adequacy of our sample size. Previous studies have confirmed that if fit indices are good, the risk of sample-related biases is significantly reduced, even in studies with smaller sample sizes [62, 63]. Although a larger sample would have provided additional statistical power, our results are aligned with contemporary psychometric standards and thus offer a reliable validation of the ESS structure. However, we had relatively small subgroup sizes, particularly among females, so the subgroup findings should be interpreted cautiously. Larger studies should be conducted to investigate whether these outcomes are robust and clinically meaningful, especially concerning gender-specific differences in EDS. Finally, a more detailed analysis of measurement invariance [64] across populations remains an important area of future research to fully assess psychometric properties of the ESS in this subset of SDB patients referred for oral appliance therapy.

## 5. Conclusions

While the dimensionality of the EDS construct measured by the ESS across populations remains open for debate, our study provides evidence supporting its unidimensionality in patients with SDB referred by physicians for oral appliance treatment. These findings are clinically relevant for dentists treating such patients and for physicians referring them, as they can continue to use and trust that a single summary ESS score accurately reflects their patients’ level of EDS. Thus, a simple sleepiness assessment based on one summary score can be considered both meaningful and reliable in this patient population.
